# Threshold Switching in Forming-Free Anodic Memristors Grown on Hf–Nb Combinatorial Thin-Film Alloys

**DOI:** 10.3390/nano12223944

**Published:** 2022-11-09

**Authors:** Ivana Zrinski, Janez Zavašnik, Jiri Duchoslav, Achim Walter Hassel, Andrei Ionut Mardare

**Affiliations:** 1 Institute of Chemical Technology of Inorganic Materials, Johannes Kepler University Linz, Altenberger Street, 69, 4040 Linz, Austria; 2 Jožef Stefan Institute, Jamova Cesta 39, 1000 Ljubljana, Slovenia; 3 Center for Surface and Nanoanalytics, Johannes Kepler University Linz, Altenberger Street, 69, 4040 Linz, Austria; 4 Danube Private University, Steiner Landstrasse 124, 3500 Krems-Stein, Austria

**Keywords:** anodic memristors, valve metals, combinatorial analysis

## Abstract

The development of novel materials with coexisting volatile threshold and non-volatile memristive switching is crucial for neuromorphic applications. Hence, the aim of this work was to investigate the memristive properties of oxides in a Hf–Nb thin-film combinatorial system deposited by sputtering on Si substrates. The active layer was grown anodically on each Hf–Nb alloy from the library, whereas Pt electrodes were deposited as the top electrodes. The devices grown on Hf-45 at.% Nb alloys showed improved memristive performances reaching resistive state ratios up to a few orders of magnitude and achieving multi-level switching behavior while consuming low power in comparison with memristors grown on pure metals. The coexistence of threshold and resistive switching is dependent upon the current compliance regime applied during memristive studies. Such behaviors were explained by the structure of the mixed oxides investigated by TEM and XPS. The mixed oxides, with HfO_2_ crystallites embedded in quasi amorphous and stoichiometrically non-uniform Nb oxide regions, were found to be favorable for the formation of conductive filaments as a necessary step toward memristive behavior. Finally, metal–insulator–metal structures grown on the respective alloys can be considered as relevant candidates for the future fabrication of anodic high-density in-memory computing systems for neuromorphic applications.

## 1. Introduction

The development of computing and information technology is progressing rapidly considering that an extensive amount of daily generated data has to be processed and stored [1,2]. Nowadays, computing systems rely on von Neumann computing architectures with separated processing and memory units [1,3]. Evidently, this can justify the fact that conventional memory technology has already reached its scaling and processing speed limits [4].

Memristors are foreseen as the most promising candidates for the next generation of non-volatile memories due to their simple structure, high switching speed, scalability, and low power consumption [2,5]. The selection of bottom and top electrodes plays a crucial role in resistive switching based on the formation of conductive filaments (CFs) inside of an active layer, this being the basis of the metal-insulator–metal (MIM) structure [6,7]. Memristors based on transitional metals have shown reliable volatile threshold and non-volatile switching characteristics [8,9,10]. While the non-volatile switching mechanism is dominated by CF formation due to the movement of O and electrolyte species inside of the oxide, threshold switching relies on the thermal formation/dissolution of metallic CFs [11,12]. The diffusive dynamics of metallic species mimic the dynamics of biological synapses in terms of synaptic plasticity regulated by Ca^2+^ dynamics. An increase or decay of the current response by applying threshold voltage values is similar to the decay of the ion concentration while releasing neurotransmitters in a neuron [13]. This emphasizes the relevance of threshold switching for a neuromorphic application but also as a selector device due to *I*–*V* nonlinearity [5,14,15,16,17,18] Synaptic weight and storage possibilities can be obtained by the implementation of non-volatile multi-level resistive switching [11,12,13]. Clearly, devices with co-existing memristive and threshold switching characteristics are desirable for the relevant applications of memristive devices [8,19,20,21,22,23]. Considering that anodic memristors have shown promising resistive switching characteristics, the further development and optimization of such devices are highly relevant [24,25,26].

Hence, the non-volatile properties of Hf memristors and threshold switching behavior, observed in Nb-based devices, were the motivation for the current study [27,28]. Additionally, it has already been observed that NbOx-based devices are forming-free since the stability of CFs can be affected by applying an electric field controlling the alignment of O species in the oxide [29,30].

Both materials, Hf and Nb, were mixed to produce bottom electrodes based on their alloys. It has been already proven that alloy-based memristors have the potential for the large-scale implementation of memristors integrated into crossbar arrays due to their stable and controllable performance. This benefits programming capabilities while consequently reducing the cost of device fabrication and broadening the range of memristive materials for neuromorphic applications [31,32]. Moreover, the selection of the active memory layer is crucial considering that it is responsible for the switching characteristics and device performance. It should be also noted that the possible future applications of these devices have to be considered when selecting the materials for an active layer [32]. Dynamic synaptic characteristics have been demonstrated for transition metal oxide (TMO) devices, including those based on HfO_2_, with these devices consuming low power and switching at numerous resistive levels [33]. Oxide-based synapses have also been used as 3D vertical-structured parallel devices meeting low-cost criteria and a high integration density [34]. Hence, this work focused on mixed oxides grown anodically on Hf–Nb alloys as active layers. Electrochemical oxidation is not only an advantageous oxide fabrication method due to its simplicity and low cost but also due to the possibility of already forming *in situ* CFs during oxide growth. In this way, forming-free memristors can be fabricated and defect engineered by the simple tuning of the electrochemical parameters [35]. This agrees with the described requirements for synaptic device optimization, while allowing controllable resistive switching in the memory layer grown on alloys [32]. Finally, the properties of anodic memristors grown on a Hf–Nb library were investigated for the first time in the current study. This is of high relevance for the development of neuromorphic systems applied for neuromorphic vision, sensors, wearable electronics, or similar [36,37,38].

## 2. Materials and Methods

### 2.1. Fabrication of Memristors

A Hf–Nb thin-film library was co-deposited by sputtering in an ultra-high vacuum system (Mantis Deposition, United Kingdom) onto thermally pre-oxidized Si wafers (950 C, 24 h). The base pressure was in the range of 10^−6^ Pa, while the deposition of Hf–Nb thin films was accomplished in DC mode at 5 × 10^−1^ Pa in an Ar atmosphere from high-purity Hf and Nb targets (99.95% Demaco Holland BV, Noord-Scharwoude, The Netherlands). The total compositional spread of the Hf–Nb system was tailored by the power levels applied to Hf and Nb targets, ranging from 25 W to 80 W, and by their positioning. More details about bottom electrode deposition by sputtering can be found elsewhere [35]. After the fabrication of bottom electrodes based on a 300 nm thick Hf–Nb library, the metallic thin films were transferred into an SEDX chamber (20 keV electron beam and spot size of 500 µm), without breaking the vacuum, for compositional analysis by a scanning energy-dispersive X-ray spectroscopy (SEDX) system connected to the same vacuum chamber cluster [39]. Following that, the electrodes were electrochemically oxidized to grow an active memristive layer. The process of anodization was conducted in a classical three-electrode system that contained an Hf–Nb system as a working electrode, a Hg/Hg_2_SO_4_/sat. K_2_SO_4_ electrode (0 V vs. Hg/Hg_2_SO_4_ = 0.640 V vs. SHE) as a reference, and a graphite foil (0.5 mm thick, 99.8 % Thermo Fisher Scientific, GmbH, Dreieich, Germany) as a counter electrode, connected to a CompactStat potentiostat (Ivium Technologies, Eindhoven, The Netherlands). The surface oxide film was grown potentiodynamically in a high-field regime by applying cyclic voltammetry to sweep the potential from 0 V to 7 V (vs. SHE) at a scan rate of 100 mV s^−1^. Citrate buffer solution (CB) was selected as the electrolyte and prepared as recommended by standard procedures for 0.1 M CB, pH = 6.0 [40]. Chemicals were of analytical grade (citric acid monohydrate (C_6_H_8_O_7_) and trisodium citrate dihydrate (C_6_H_9_Na_3_O_7_)) and used as purchased (Merck, KGaA, Darmstadt, Germany).

A typical metal–insulator–metal (MIM) structure was completed by top electrode patterning through a pre-attached Ni shadow mask foil (Mecachimique, Pierrelaye, France) by sputtering from a Pt target (99.95% Demaco Holland BV, Noord-Scharwoude, The Netherlands) at room temperature. In total, approximately 300 memristors (grouped in 5 × 5 electrode clusters within each 3 × 3 mm^2^ surface area) were produced.

### 2.2. Electrical Characterization of Memristive Devices

Using a combinatorial approach, various electrical characteristics could be examined at the same time for memristors based on different alloys. The electrical screening of the Hf–Nb system was conducted using a Gantry robot connected to a source meter (Keithely 2450). Such a system was self-developed and controlled via LabView^®^ software specifically programmed for typical memristive characterization experiments (*I–U* sweeps, endurance, and retention measurements). The setup is described in detail in previous studies [27,41]. All measurements were performed in environmental conditions (22 °C, 55% RH) while contacting the top electrodes with a W needle (10 µm tip diameter) with a constant force kept at 20 ± 2 mN and the bottom electrodes by a stainless-steel needle. The metallic thin films on Hf–Nb were polarized, whereas the Pt electrodes remained grounded. The pulsed voltage stress (PVS) approach was used to test the endurance of the devices at a frequency of 260 Hz while limiting the current up to 10 Ma. The data retention was investigated by reading the resistance values for the current corresponding to a polarization voltage of 10 mV in identical conditions. In both cases, up to 10 devices were tested for each alloy indicating memristive behavior from *I–U* sweeps.

### 2.3. Microscopic and Spectroscopic Analysis

The samples for transmission electron microscopy (TEM) were prepared by a site-specific focused ion beam technique (FIB, Helios NanoLab 650i, FEI BV, Eindhoven, The Netherlands). The thin lamella was cut from a selected memristor sample, welded onto a Cu support, and thinned by Ga ions until a final thickness of <100 nm. The microstructure and crystallography analyses were performed by conventional TEM (JEM-2100, JEOL Inc.) and scanning TEM (STEM, ARM-200CF, JEOL Ltd., Tokyo, Japan), both operating at 200 kV.

A Theta Probe system (Thermo Scientific, East. Grinstead, UK) was used for XPS measurements. The system was controlled by the Avantage software (version 5.9925) package provided by the system manufacturer (Thermo Scientific, East Grinstead, UK). The samples were analyzed with monochromatic Al Kα X-ray radiation (1486.6 eV), being focused into 400 μm diameter spots. Survey spectra were acquired with a pass energy of 200 eV and a binding energy step of 1 eV. A pass energy of 20 eV with an energy step of 0.05 eV was applied for the high-resolution spectra. A dual flood gun was used for the charge compensation accumulated on the surface. The measured spectra were calibrated to the C1s peak of the adventitious carbon found to be at 285.0 eV.

## 3. Results

### 3.1. Combinatorial Screening of Memristive Behavior in Hf–Nb System

The relevance of the combinatorial approach as well as the simplicity of thin-film library fabrication has been already confirmed in many previous studies [42,43,44,45,46]. However, only recently has the combinatorial screening of memristive properties been proven as a useful tool for the identification of high-performance memristors [35,47]. This is crucial for the development of anodic memristors with the potential for industrial implementation, assuming a large amount of data is collected within only one library scan. In this respect, the memristive and electrical characteristics of the anodized Hf–Nb system, with a total compositional spread of 74 at.%, were investigated for the first time in this work. Alloys with compositions ranging from Hf–18.5 at.% Nb to Hf-92.5 at.% Nb were spread over three pre-oxidized Si wafers. The properties of these alloys, with the amount of Hf ranging from 7.5 at.% to 81.5 at.%, were compared with the properties of pure Nb and Hf thin films, these representing reference samples. Based on studies of memristors grown on pure metals in different electrolytes, citrate buffer solution was selected for the anodization since both Hf and Nb memristors exhibited the best performance in the respective solutions [27,28].

As described in the experimental section, a lateral compositional resolution in the range of 0.2 at.% mm^−1^ was calculated for the entire Hf–Nb library based on SEDX mappings. Nevertheless, the compositional precision was estimated to be 1 at.% assuming the assessed error of the SEDX analysis, thus defining each alloy by one cluster of 5 × 5 electrodes on a surface of 5 × 5 mm^2^. Taking into account that each cluster represented one alloy, more than 200 clusters, each with 25 devices, were analyzed on each Si substrate. Hence, a systematic screening along the compositional gradient and perpendicular to it was relevant to identify the devices based on alloys with different or identical compositions, respectively. This allowed the extraction of cycle-to-cycle and device-to-device variabilities, which are recommended parameters for studying memristive behaviors [48].

Unlike previous studies based on different binary systems, the memristors found in the Hf–Nb library could not be grouped in compositional zones according to their common memory or electrical characteristics [35,47]. No regular trend in memristive properties was observed along the compositional gradient. Nonetheless, the devices based on several specific alloys demonstrated improved behaviors as compared to the anodic devices based on pure metals, as is discussed further.

### 3.2. General Micro- and Nano-Scale Characterization

**Figure 1:**
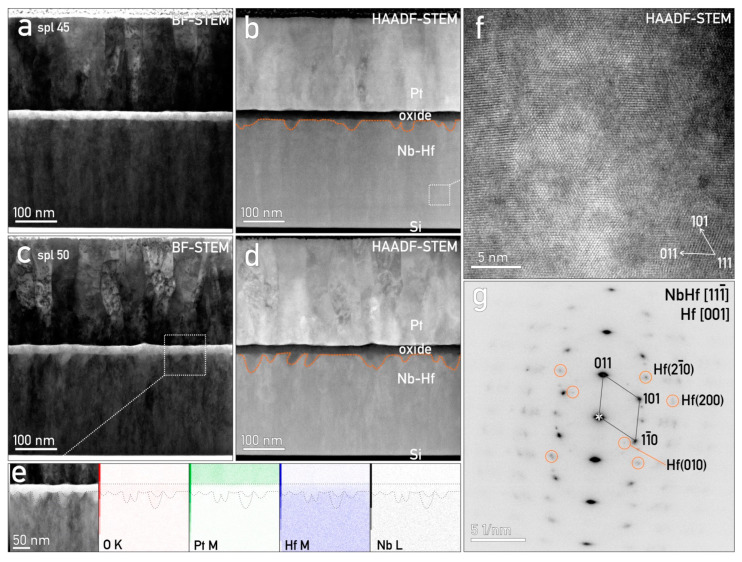
General outline of the phases present in the sample Nb-45 at.% Hf and Nb-50 at.%Hf. (**a**,**c**) BF-STEM and (**b**,**d**) HAADF-STEM micrographs of top Pt electrode with columnar structure, intermediate oxide layer, and underlying Hf–Nb metal. (**e**) EDX map of principal chemical components, where both Nb and Hf were present in the oxide layer; (**f**) bulk Hf–Nb showed preferential pillar-like growth with nano-scaled Hf exsolutions. (**g**) SAED of same region, indexed for Hf–Nb and (encircled) for Hf.

The overall MIM structures grown on the Hf–Nb library system are visible in Figure 1 as formed on the Si wafer covered by 500 nm of amorphous SiO_2_. The thickness of the Hf–Nb metallic thin film was in good agreement with the initial 300 nm estimation (from the sputtering conditions), whereas the anodic oxide was approximately 20 nm thick. The stack was covered by a 250 nm crystalline Pt top electrode with a columnar structure (Figure 1a,b). On the one hand, no voids or layer spallation on the interfaces were observed. The interface between the SiO_2_ and Hf–Nb alloy was sharp and well-defined. On the other hand, the alloy–oxide interface seemed uneven, and the oxide film appeared to occasionally protrude into the metal (marked with a dashed line in Figure 1c). This may be related to the anodization process, where both Hf and Nb ions originating from the alloy competed for O. This dynamic responsible for the formation of these protrusions was especially enhanced here since species producing both crystalline and amorphous oxides were used [42]. The transition between oxide and Pt was sharp, and the Pt layer followed the topography of the underlying oxide and alloy. The Pt grains/columns extended throughout the whole thickness of the layer, being about 50 nm wide and preferentially oriented in the <110> direction (Figure 1a–d). The chemical analysis by EDX of the layers presented in Figure 1e revealed that both Nb and Hf were present in the oxide layer. The Pt covering electrode did not protrude into the oxide or vice versa. The oxidation of the Hf–Nb metallic thin film resulted in an uneven 3D surface, which contributed to small thickness variations in the oxide layer. The Nb–M line overlapped the Pt-M line, hence the less intensive Nb-L characteristic energy peak was used for visualization.

Variation in the phase contrast on the high-resolution micrographs indicated a certain chemical inhomogeneity of the Hf–Nb alloys (Figure 1f), likely resulting from the atomically mixing deposition process. The analysis of the crystal structure via selected area electron diffraction (SAED) patterns showed the existence of a cubic Hf–Nb alloy, confirming previous studies [42]. The measured d-values, if following Vegard’s law, corresponded to a Hf-depleted mixture when compared to the initial composition. Indeed, when carefully examining the SAED pattern, one could identify additional weak subordinate diffraction peaks, corresponding to hcp Hf (Figure 1g). Such nano-scaled oriented Hf domains were not precipitates, but most likely resulted from the alloy deposition procedure.

### 3.3. XPS Analysis

**Figure 2:**
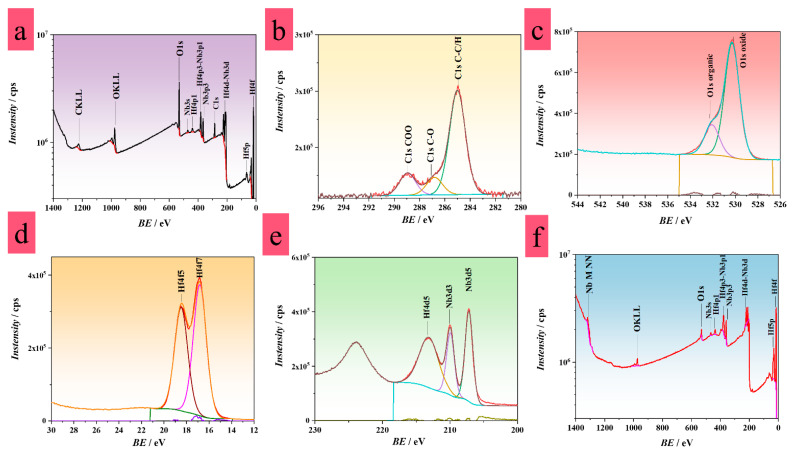
(**a**,**f**) XPS survey and (**b**–**e**) high-resolution spectra of Hf-45 at.% Nb samples prepared in CB.

The composition of the oxide grown on Hf-45 at.% Nb was investigated by XPS (Figure 2). The presence of C, O, Hf, and Nb was confirmed at the surface of the anodic oxide grown in CB. As visible in Figure 2b, three chemical forms of C were identified from the C 1s high-resolution spectra. The adventitious C was related to the C-C/C-H carbon type, with a peak at 285.0 eV. The second peak found at 288.9 eV was assigned to a carboxylic functional group (O=C-O), while the peak at 286.8 eV stood for carbon bonded with a single covalent bond to oxygen (C-O) [49,50]. Both, the O=C-O- and C-O-like carbon peaks were attributed to a citrate ion. Similarly, two different chemical forms of O were distinguished based on the O 1s spectrum (Figure 2c). These forms were assigned to peaks at 532.0 eV and 530.3 eV as O bound in oxide and O from the carboxylic group, respectively [49,50,51]. A single chemical form of Hf is visible in Figure 2c as a Hf 4f scan. Furthermore, the Hf 4f_7/2_ peak at 16.8 eV represents its oxide, HfO_2_. The same refers to peaks found at 213.1 eV (Hf 4d_5/2_) and at 18.5 eV (Hf 4f_5/2_) in Figure 2 [51]. This confirmed a stoichiometric amount of O to Hf. In the case of Nb, the Nb 3p scan described in Figure 2a,e specifies a single form of Nb, while peaks detected at 209.9 eV (Nb 3d_3/2_) and 207.2 eV (Nb 3d_5/2_) indicate an oxidized Nb form as Nb_2_O_5_ [52]. A shift of BE was observed with increasing probing depth as shown in the spectrum in Figure 2f. Peaks positioned at 13.44 eV (Hf 4f_7/2_) and at 201.9 eV (Nb 3d_5/2_) identified single forms of Hf and Nb, respectively [53,54]. This determined a nominal bulk composition of the alloy as Hf-43 at.% Nb, confirming the initial EDX measurements. Additionally, the BE shift of the Nb 3d_3/2_ peak at 204.6 eV was indicative of NbO_2_ [52]. Hence, the formation of oxide in CB may have resulted in different oxide stoichiometries at the surface and in the bulk of the oxide. This was further linked to the switching characteristics of the memristors grown of Hf-45 at.% Nb exhibiting a forming-free threshold and non-volatile behavior at the same time [55,56]. It may be assumed that the NbO_2_ layer, responsible for threshold behavior, acted as an interfacial layer between the reactive bottom electrode and the mixed oxide that increased the selectivity of NbO_2_ towards O species, thus supporting the observed CFs stability [29].

### 3.4. Memristive Switching of Anodized Hf–Nb Library

**Figure 3:**
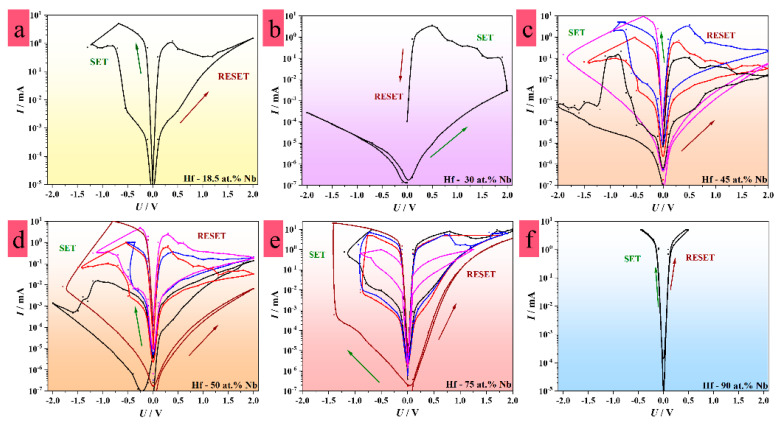
Representative memristive switching hysteretic *I*–*U* curves plotted in logarithmic current scale for selected memristors defining different memristive characteristics along the Hf-Nb combinatorial library: (**a**) bipolar switching characteristics of MIM structures grown on Hf-enriched alloys, (**b**) unipolar switching characteristics of MIM structures grown on Hf-enriched alloys, (**c**,**d**) unipolar and bipolar reversible threshold switching for high-performing MIM structures grown on Hf-45 at.% Nb and Hf-50 at.% Nb alloys, (**e**,**f**) irregular switching characteristics for MIMs grown on Nb-enriched alloys.

Memristive switching in the Hf–Nb system was investigated by performing *I–U* sweeps for 25–100 devices defined for a specific alloy. The maximum voltage sweeping from −3 V to 3 V was conducted under current limitations in the range from 0.1 mA to 30 mA to prevent irreversible switching to LRS. The sweeping is exemplified in Figure 3 for the anodic oxides grown on different Hf–Nb alloys. The metal–insulator–metal structures showing memristive characteristics were forming-free, suggesting low power consumption since it was not necessary to apply additional voltage to form the CFs. In addition, forming-free memristors are desirable since a CFs size and positioning are predictable due to their intrinsic formation during the anodization process [35,47]. Additionally, the ratio between HRS and LRS values (each measured from the slopes of linear parts in the *I*–*U* sweeps) defines an important characteristic describing the memory window and expected power consumption of a device [6]. Accordingly, the higher the difference between resistive states, the larger the memory window and the lower the power consumption.

As can be seen in Figure 3, typical hysteresis loops demonstrating memristive behavior were exemplified for the different alloys along the entire library. Additionally, the supplemental Figure S1 presents representative *R*–*U* sweeps. The devices grown on alloys containing a low amount of Nb (exemplified by Hf-18.5 at.% Nb in Figure 3a) showed bipolar threshold switching characteristics with resistive state ratios (HRS/LRS) up to 100. The resistive state ratio increased with the amount of Hf in the alloys, reaching seven orders of magnitude at much higher Hf contents. However, a transition was observed for the alloys around Hf-30 at.% Nb, which showed unipolar switching in the positive direction (Figure 3b). At the same time, applying different current limitations did not result in switching at different resistive levels. Multi-level switching characteristics are one of the most important parameters for neuromorphic applications due to the possibility of building high-density memory [57]. Increasing the Nb concentration above 30 at.% resulted in the recovery of the bipolar switching behavior, as exemplified in Figure 3c, where the memristive behavior was enhanced. The devices grown on Hf-45 at.% Nb alloys showed bipolar threshold switching characteristics (Figure 3c) at several resistive levels. The observed bipolar–unipolar–bipolar transition at around 30 at.% Nb was likely related to the structural and electrical characteristics of the mixed anodic oxides grown in this region [35]. It has previously been reported that both the oxide formation factor and dielectric constant show a maximum in the vicinity of the Hf-30 at.% Nb [42]. Since the dielectric constant was related to the polarizability of the mixed oxides (which may directly affect the formation of CFs and the switching mechanism), it was likely responsible for the unipolar behavior of the oxides grown on this particular Hf–Nb alloy [35,42].

Similarly to Hf-45 at.% Nb, the devices formed on the Hf-50 at.% Nb alloys demonstrated threshold switching up to five different resistance levels (Figure 3d). The resistive state ratio reached 10^4^ and 10^6^ in the case of the Hf-50 at.% Nb and Hf-45 at.% Nb alloys, respectively. This already indicated the improved performance of the devices grown on these alloys in comparison to devices grown on the alloys with other compositions. The switching characteristics of the devices formed on the alloys containing Hf-75 at.% Nb showed similar features in the low-voltage range (Figure 3e). However, different resistive switching levels were not distinguishable. Hence, the switching at different resistance levels could not be fully controlled by applying different current compliances in the devices formed on the Hf-50 at.% Nb alloys, as opposed to the devices grown on Hf-45 at.% Nb. Additionally, the highest amount of Nb in the alloys resulted in devices with a low HRS/LRS ratio (Figure 3f).

**Figure 4:**
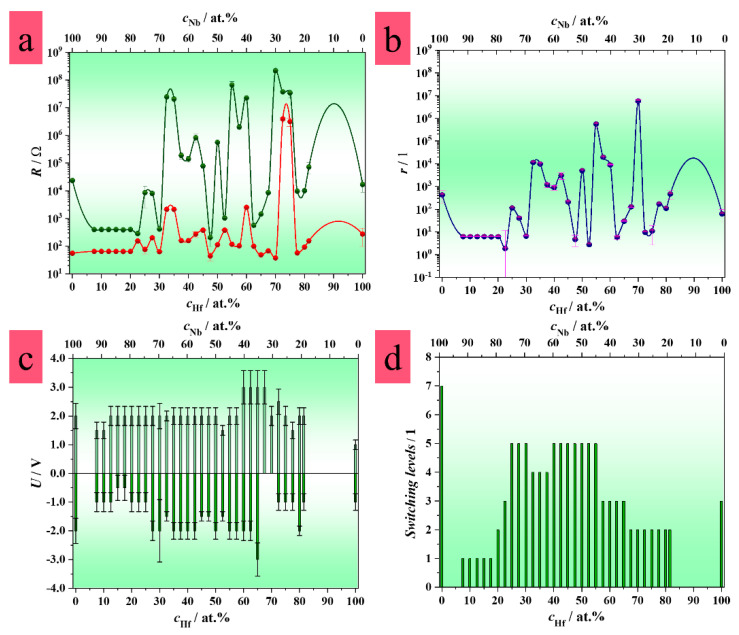
Compositional mapping in the Hf-Nb library system describing anodic memristors: (**a**) high and low resistive state values, (**b**) resistive state ratio, (**c**) switching voltage values, (**d**) number of switching levels.

The trend of important electrical and memory characteristics with the respect to the total compositional spread of the Hf–Nb system is presented in Figure 4. The resistive state ratio increased with the amount of Hf in the alloys, with a maximum for the composition of Hf-30 at.% Nb (Figure 4a,b). However, the devices grown on Hf-30 at.% Nb did not show high reproducibility regarding their lifetime and data retention, which are important parameters, as is discussed further. A switching voltage range of up to 2 V was found for these MIM structures in addition to irreversible switching from the unipolar to bipolar mode. This behavior was further observed for the alloys containing between 30 and 40 at.% Nb in a wider switching voltage range from −3 V to 3 V (Figure 4c). In contrast, the MIM structures formed on the alloys containing more than 45 at.% Nb switched in a lower voltage range (Figure 3 and Figure 4c). Moreover, a maximum of five different switching levels were achieved for the MIM structures grown on the alloys containing between 45 and 75 at.% Nb, which represented an improvement in comparison with the anodic memristors formed on pure Hf [27,58].

In addition to the switching at several resistive levels, the common characteristic of all the devices was threshold switching. In this case, typically one state is volatile, while the other resistive state behaves as non-volatile. The switching to a HRS or LRS state, defining the RESET or SET process, respectively, is automatically induced by removing the externally applied electric field [48]. The coexistence of non-volatile and volatile behavior has already been reported for active layers based on Nb, which depends on the O content. While Nb_2_O_5_ shows memristive switching, threshold switching was evidenced for devices with NbO_2_ [22,59,60,61]. On the one hand, threshold behavior is commonly observed for transition metals due to thermally induced insulator-to-metal transition (IMT) [62,63,64,65]. On the other hand, non-volatile memristive behavior is related to CF formation and rupture, their number, and their size [2,41]. Taking into account these facts, it may be assumed that the threshold switching of the current Hf–Nb system was influenced by Nb and O species contained in the mixed oxides of the analyzed devices. Following that, Hf and the incorporated electrolyte species may have affected the non-volatile behavior, as confirmed in previous investigations. Hence, the formation of CFs was directly dependent upon the composition of the alloys [9]. Enhanced memristive performances, such as the highest resistive ratio and lowest controllable switching voltages at several resistive levels, (Figure 4b–d) suggested that a slightly higher amount of Hf in the alloys may have favored the stability of the CFs. This was exemplified by the metal–insulator–metal structures grown on the alloys at Hf-45 at.% Nb and Hf-50 at.% Nb presented in Figure 3c–d. The memristive effect, based on metallic CF formation/rupture (see Section 3.6), was also likely supported by the negative differential resistance (NDR) effect. This was defined by the current density decreasing with the increase in the electric field (Figure 3a). Memory effects accompanied by the NDR effect have already been demonstrated for devices operating at room temperature, showing potential for the development of multifunctional electronics [66]. The co-occurrence of memory and the NDR effect is related to different conduction mechanisms such as Schottky emissions, hopping, direct tunneling, and Ohmic conduction. Hence, the switching mechanism can be also discussed by fitting the typical *I–U* sweeps using these conduction models. In addition, photovoltaic theory suggests the movement of electrons and holes according to the different work functions of different electrode materials [66]. Therefore, the switching mechanism is a synergy of different effects and conduction mechanisms as already reported in previous research [27]. Not only were multi-level switching tests performed by applying different current compliances (*I*_cc_), but also the switching modes were studied. It was observed that unidirectional or bidirectional switching depended upon the current limitation range (Figure 3b–d). Generally, the memristors grown on the different alloys showed bidirectional switching when applying an *I*_cc_ up to 5 mA, whereas unidirectional switching appeared for an *I*_cc_ in the range of 10 mA or higher. Switching in a positive direction was observed for the memristors grown on Hf-30 at.% Nb (Figure 3b), while the memristive devices formed on the alloys containing more than 45 at.% Nb switched in the negative direction for a current limitation set at 10 mA (Figure 3c,d and Figure 4c). Generally, the MIM structures grown on Nb enriched alloys, switched in a bipolar mode for current compliances up to 10 mA. Hence, only the bipolar switching characteristics for the memristive devices formed on the alloys with more than 45 at.% Nb are shown in Figure 4c. Such observations were easily explained by the fact that unipolar switching is a thermally induced process in which the dissolution of defects or local oxidation/reduction appears due to Joule heating induced by an electric field, thus causing the formation/disruption of CFs in a violent manner. The ionic drift of O or electrolyte-based species through an oxide induces electrochemical redox reactions allowing the formation of CFs, which defines the bipolar switching mode [67]. Since more heat is generated under a higher *I*_cc_ regime, unipolar switching is more likely to appear than bipolar switching, as was detected herein [68]. The switching mechanism will depend upon the voltage polarity. As can be seen in Figure 3, the switching to LRS (SET process) was achieved by applying voltages up to the threshold voltage value of negative (or positive) polarity, when mobile species drifted to the cathode interface where the reduction of CFs appeared. Oppositely, when applying positive (or negative) voltage values or lower than the hold voltage values, the species drifted toward the anode interface, leading to the oxidation of CFs, thus switching to HRS (RESET process). This additionally confirmed the threshold switching behavior, in which a gate voltage value (threshold voltage) should be reached to switch a device from HRS to LRS. Oppositely, values lower than the hold voltage (the minimum voltage necessary to be applied for a device to maintain a current resistive state), should be reached to switch the device back to HRS. In this case, HRS is described as volatile, since reaching a voltage of 0 V defines the RESET process [69]. In other words, by removing an external electric field the memristor is spontaneously switched to HRS.

### 3.5. Electrical Characteristics of Hf–Nb Anodic Memristors

**Figure 5:**
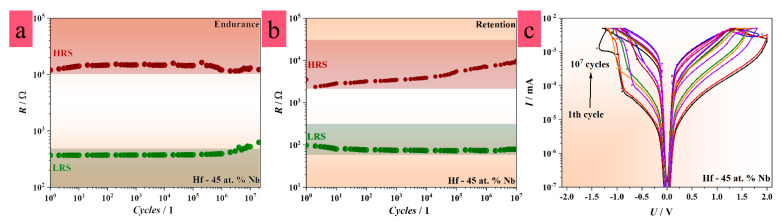
(**a**,**b**) Endurance and retention measurements recorded for MIM structures with improved memristive properties, (**c**) *I*–*U* sweeps recorded during consecutive writing procedure (endurance measurements).

As already mentioned, the device lifetime and data retention were investigated by performing endurance and retention tests for up to 10 MIM structures formed on the alloys indicating memristive behavior. The switching voltages, determined during *I–U* sweeping, were applied in both cases. Cycle-to-cycle and device-to-device variabilities were empirically assessed from the measurements for a given alloy. The representations of the statistical range for both cycle-to-cycle and device-to-device variabilities were conducted by the colored confidence bands as shown in Figure 5 and Figure S2.

In general, no relevant differences in device lifetime and data retention were detected for the devices based on the alloys exhibiting memristive behavior. However, the memristors grown on the alloys with either a low or high content of Hf did not reach a reasonable number of writing or reading cycles (maximum 100 cycles). The memristors grown on Hf-45 at.% Nb and Hf-50 at.% Nb demonstrated improved performance, while those grown on Hf-75 at.% Nb showed significant lifetime and data retention capabilities (see Supplemental Figure S2a,b), especially in comparison with the reference samples based on pure metallic films. The endurance and retention measurements for the MIM structures on Hf-45 at.% Nb and Hf-50 at.% Nb reached more than 10^7^ cycles, as presented in Figure 5a,b. Typical *I–U* sweeps recorded after each cycles order of magnitude during the writing procedure are shown in Figure 5c. It should be noted that an HRS/LRS ratio smaller than 10 is considered the end of the device lifetime. As is visible in Figure 5, the HRS values became gradually more conductive, while the LRS values became more insulating during the writing process. Oppositely, the LRS values decreased, while the HRS values further increased during the reading process. This was explained by O depletion at the oxide/electrode interfaces as a result of stronger or weaker CF formation due to the continuous loss of O to the bottom and top electrodes [70]. Thus, the accumulation of O or O vacancies appeared at both electrode interfaces simultaneously. Accordingly, an increased concentration of O vacancies in the oxide, thickening the CFs or the sensitivity of the CFs to thermal disturbances led to retention failure (Figure 3b) [71]. In addition, cycle-to-cycle and device-to-device variabilities were mild in comparison to the devices based on the alloys with high or low content of Hf (Figure 3a,b,f). Both variabilities even reached a few orders of magnitude for LRS and HRS (Figure S2a,b), whereas the variabilities for the devices grown on Hf-45 at.% Nb and Hf-50 at.% Nb were up to one order of magnitude (Figure 5a,b). Since these devices switched at several resistive levels, the variabilities are shown only for the writing and reading procedures recorded at current compliances of 5 mA due to the simplicity of the representation. Overall, the device lifetime, memory, and cycle-to-cycle and device-to-device variabilities were likely affected by the CF size, shape, number, and positioning [27,28,35,41,58].

### 3.6. Nanostructural Characterization of Hf–Nb Anodic Memristors and CF Formation

**Figure 6:**
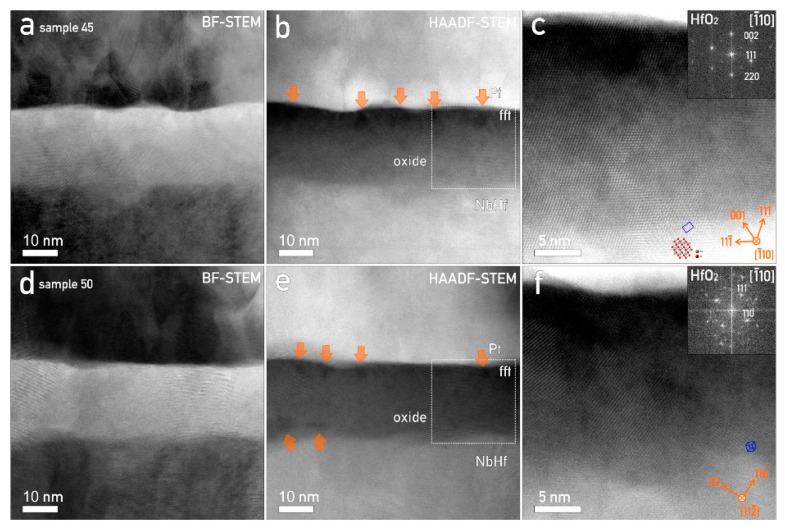
Nanostructural characterization of the Hf–Nb oxide layer, top: sample Hf-45 at.% Nb and, bottom: sample Hf-50 at.% Nb. (**a**,**d**) BF-STEM and (**b**,**e**) HAADF-STEM micrographs of crystalline and amorphous oxide layer; orange arrows mark amorphous regions. (**c**) Sample Hf-45 at.% Nb: large HfO_2_ crystallites extending through whole width of the oxide layer. (**f**) Sample Hf-5 at.% Nb: smaller HfO_2_ randomly oriented crystallites.

The best-performing samples from the Hf–Nb library (the Hf-45 at.% Nb and Hf-50 at.% Nb alloys) were selected for TEM analysis to characterize the nanostructure of their oxides. By employing STEM, a detailed view showed that the oxide layer formed between the Hf–Nb thin film and the Pt top electrode was a combination of amorphous and crystalline structures (Figure 6). The identification of crystalline HfO_2_ was based on HAADF-STEM atomic-scale micrographs (Figure 6c). The amorphous phase inside of the oxide was related to darker accumulation regions as visible in Figure 6b. Such regions formed at the surface of the oxide were due to its anodic growth in the citrate buffer solution affecting the CF formation [27,28].

It was also found that the crystallites were larger, extending throughout the oxide, for the samples grown on Hf-45 at.% Nb (Figure 6a–c) as compared to those of the oxide grown on the Hf-50 at.% Nb alloys. In the last case, only several randomly oriented crystals were detected (Figure 6d–f).

**Figure 7:**
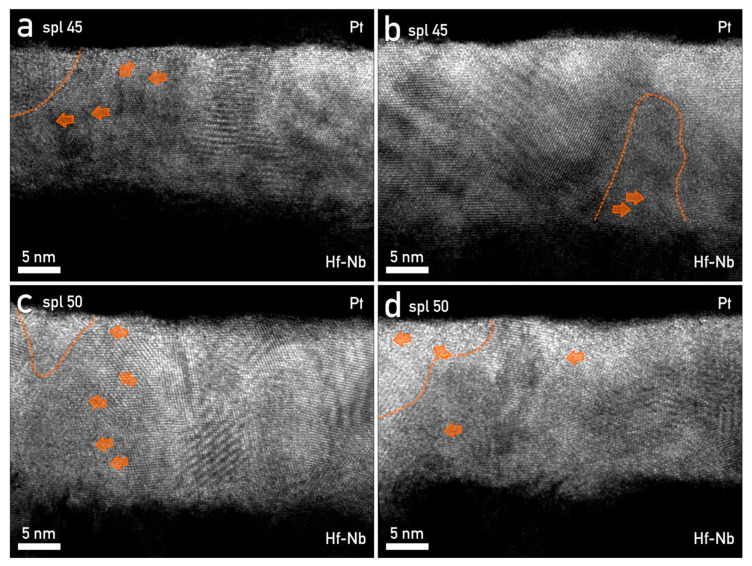
HR-TEM micrographs of Hf–Nb oxide layer. (**a**,**b**) Sample Hf-45 at.% Nb, (**c**,**d**) sample Hf-50 at.% Nb. Crystalline HfO_2_ was sporadically interrupted by amorphous Nb_2_O_5_ regions (outlined with a dashed orange line). The conductive filaments emerging from metal layers are marked by orange arrows.

As already mentioned in the previous sections, the structure of anodic oxides plays a crucial role in resistive switching due to CF formation/rupture inside of it. These events are not only associated with the oxide structure but also with the structure of the electrode material and the species incorporated in the oxide [68]. Figure 7 illustrates the high-resolution mixed anodic oxides grown on the Hf-45 at.% Nb and Hf-50 at.% Nb alloys. Crystallites of HfO_2_ were seen as embedded throughout the entire mixed oxide. These HfO_2_ crystallites were surrounded by a mixed anodic oxide matrix, which was in contact with nano-scale oriented Hf domains (Figure 7a–d). Nevertheless, amorphous Nb_2_O_5_ regions and inhomogeneities (CFs) were identified and are marked by orange dashed lines and arrows, respectively. Similar structures have already been reported in previous memristive studies when mixing amorphous and crystalline oxides [35]. Such regions may be favorable for CF positioning, ensuring their constant number and size. Consequently, this may benefit the device lifetime, retention, and lower cycle-to-cycle as well as device-to-device variabilities. Such regions may also explain the unipolar resistive switching mode, this being also characteristic in this case for an I_cc_ of 10 mA (Figure 1c) [27,28,35,41,58]. 

The co-existence of unipolar and bipolar switching modes was justified by the presence of HfO_2_ crystallites embedded in the oxide matrix, as well as several CFs partially connecting the bottom and top electrodes, as seen for the MIM structure grown on the Hf-45 at.% Nb and Hf-50 at.% Nb alloys (Figure 7). The formation of HfO_2_ crystallites during the anodization process confirmed that forming-free memristors could be grown while improving their performance due to the enhanced stability of the CFs positioned in such regions [27,35]. Conductive filament concurrency has already been observed for anodic memristors grown on pure Hf thin films, in which non-disrupted, disrupted, and amorphized CFs were imaged in the oxide [27,35]. Their formation or rupture was dictated by O vacancy accumulation regions at the Pt/oxide interface (Figure 7c), while their spatial pinning was justified by the incorporation of electrolyte species [27]. Additionally, the coexistence of a forming-free threshold and non-volatile resistive switching was explained by the non-uniformity of the oxides in terms of stoichiometry, as has also been proven in previous reports [28]. Typically, device-based non-stoichiometric Nb oxides demonstrate volatile threshold switching, while non-volatile resistive switching is usually observed for devices based on stoichiometric Nb oxides [55,56]. Moreover, the presence of NbO_2_ at the bottom electrode/oxide interface, confirmed by XPS, could additionally explain the fact that these memristors were forming-free. It has already been reported that a Nb(O) electrode can adsorb O, thus depleting the NbOx layer via an applied electric field. In such a case, an active layer based on NbOx is defined as a mixture of insulating Nb_2_O_5_ and conducting NbO_2_. Hence, the forming-free threshold memristive switching is affected by the alignment of conductive phases when applying an electric field or by the alignment of O vacancies within the oxide impacting CF formation [29,30].

## 4. Conclusions

Following the screening results of the anodic memristors grown on a Hf–Nb combinatorial library, the coexistence of threshold and non-volatile memristive switching was observed for metal–insulator–metal structures grown on Hf-45 at.% Nb and Hf-50 at.% Nb alloys. At the same time, improved performances regarding resistive state ratios, low voltage switching ranges, or multi-level switching characteristics, were demonstrated for the respective MIMs. In addition, the memristive switching in the bipolar or unipolar mode was identified to be dependent upon the current compliance applied during the I–U switching. Unipolar switching characteristics were found at a higher current compliance range, while bipolar ones were evidenced at a lower current compliance range. This was explained by Joule heating typically released for the higher current compliance range. The mixed switching behavior for the MIMs grown on the Hf-45 at.% Nb and Hf-50 at.% Nb alloys was also justified by HfO_2_ crystallites embedded in the mixed oxides, facilitating the formation of CFs and was related to the non-uniformity of the Nb oxides’ stoichiometry. Finally, a regular trend in resistive switching could not be recognized by the high-throughput screening of the anodized Hf–Nb system. Nevertheless, the screening outcome indicated that MIMs grown on Hf-45 at.% Nb and Hf-50 at.% Nb alloys may be considered for the fabrication of high-density in-memory computing systems for neuromorphic applications.

## Data Availability

The data presented in this study are available on request from the corresponding author.

## Supplementary Materials

The following supporting information can be downloaded at: https://www.mdpi.com/article/10.3390/nano12223944/s1, Figure S1: Representative *R*–*U* sweeps along the Hf-Nb anodic memristors. Figure S2: (a,b), Endurance and retention measurements recorded for metal-insulator-metal (MIM) structures grown on Hf-75 at.%Nb alloys, (c) *R*–*U* sweeps for MIMs formed on Hf-50 at.%Nb, (d) representative *I*–*U* sweep recorded for MIMs formed on Hf-50 at.%Nb alloys.

## References

[1] Im I.H., Kim S.J., Jang H.W. (2020). Memristive Devices for New Computing Paradigms. Adv. Intell. Syst..

[2] Sun K., Chen J., Yan X. (2021). The Future of Memristors: Materials Engineering and Neural Networks. Adv. Funct. Mater..

[3] Sebastian A., Le Gallo M., Khaddam-Aljameh R., Eleftheriou E. (2020). Memory devices and applications for in-memory computing. Nat. Nanotechnol..

[4] Nishi Y. (2014). Advances in Non-Volatile Memory and Storage Technology.

[5] Upadhyay N.K., Jiang H., Wang Z., Asapu S., Xia Q., Joshua Yang J. (2019). Emerging Memory Devices for Neuromorphic Computing. Adv. Mater. Technol..

[6] Ielmini D. (2016). Resistive switching memories based on metal oxides: Mechanisms, reliability and scaling. Semicond. Sci. Technol..

[7] Yang J.J., Strukov D.B., Stewart D.R. (2013). Memristive devices for computing. Nat. Nanotechnol..

[8] Abbas H., Abbas Y., Hassan G., Sokolov A.S., Jeon Y.R., Ku B., Kang C.J., Choi C. (2020). The coexistence of threshold and memory switching characteristics of ALD HfO2memristor synaptic arrays for energy-efficient neuromorphic computing. Nanoscale.

[9] Sun Y., Song C., Yin S., Qiao L., Wan Q., Wang R., Zeng F., Pan F. (2020). Design of a Controllable Redox-Diffusive Threshold Switching Memristor. Adv. Electron. Mater..

[10] Li H.Y., Huang X.D., Yuan J.H., Lu Y.F., Wan T.Q., Li Y., Xue K.H., He Y.H., Xu M., Tong H. (2020). Controlled Memory and Threshold Switching Behaviors in a Heterogeneous Memristor for Neuromorphic Computing. Adv. Electron. Mater..

[11] Li Y., Tang J., Gao B., Sun W., Hua Q., Zhang W., Li X., Zhang W., Qian H., Wu H. (2020). High-Uniformity Threshold Switching HfO2-Based Selectors with Patterned Ag Nanodots. Adv. Sci..

[12] Wang Z., Rao M., Midya R., Joshi S., Jiang H., Lin P., Song W., Asapu S., Zhuo Y., Li C. (2018). Threshold Switching of Ag or Cu in Dielectrics: Materials, Mechanism, and Applications. Adv. Funct. Mater..

[13] Xiao M., Shen D., Futscher M.H., Ehrler B., Musselman K.P., Duley W.W., Zhou Y.N. (2020). Threshold Switching in Single Metal-Oxide Nanobelt Devices Emulating an Artificial Nociceptor. Adv. Electron. Mater..

[14] Minnekhanov A.A., Emelyanov A.V., Lapkin D.A., Nikiruy K.E., Shvetsov B.S., Nesmelov A.A., Rylkov V.V., Demin V.A., Erokhin V.V. (2019). Parylene Based Memristive Devices with Multilevel Resistive Switching for Neuromorphic Applications. Sci. Rep..

[15] Burr G.W., Shelby R.M., Sebastian A., Kim S., Kim S., Sidler S., Virwani K., Ishii M., Narayanan P., Fumarola A. (2017). Neuromorphic computing using non-volatile memory. Adv. Phys. X.

[16] Sokolov A.S., Abbas H., Abbas Y., Choi C. (2021). Towards engineering in memristors for emerging memory and neuromorphic computing: A review. J. Semicond..

[17] Zhang X., Huang A., Hu Q., Xiao Z., Chu P.K. (2018). Neuromorphic Computing with Memristor Crossbar. Phys. Status Solidi Appl. Mater. Sci..

[18] Wang Z., Joshi S., Savel’ev S.E., Jiang H., Midya R., Lin P., Hu M., Ge N., Strachan J.P., Li Z. (2017). Memristors with diffusive dynamics as synaptic emulators for neuromorphic computing. Nat. Mater..

[19] Li Y., Yuan P., Fu L., Li R., Gao X., Tao C. (2015). Coexistence of diode-like volatile and multilevel nonvolatile resistive switching in a ZrO_2_/TiO_2_ stack structure. Nanotechnology.

[20] Liu X., Md Sadaf S., Son M., Park J., Shin J., Lee W., Seo K., Lee D., Hwang H. (2012). Co-occurrence of threshold switching and memory switching in Pt/NbO x/Pt cells for crosspoint memory applications. IEEE Electron Device Lett..

[21] Abbas H., Ali A., Jung J., Hu Q., Park M.R., Lee H.H., Yoon T.S., Kang C.J. (2019). Reversible transition of volatile to non-volatile resistive switching and compliance current-dependent multistate switching in IGZO/MnO RRAM devices. Appl. Phys. Lett..

[22] Mahne H., Wylezich H., Slesazeck S., Mikolajick T., Vesely J., Klemm V., Rafaja D. Room temperature fabricated NbOx/Nb_2_O_5_ memory switching device with threshold switching effect. Proceedings of the 2013 5th IEEE International Memory Workshop 2013.

[23] System N.N.X., Luo Q., Zhang X., Member S., Yu J., Wang W. (2019). Memory Switching and Threshold Switching in a 3D nanoscaled NbO x system. IEEE Electron Device Lett..

[24] Diamanti M.V., Pisoni R., Cologni A., Brenna A., Corinto F., Pedeferri M.P. (2016). Anodic oxidation as a means to produce memristive films. J. Appl. Biomater. Funct. Mater..

[25] Kundozerova T.V., Grishin A.M., Stefanovich G.B., Velichko A.A. (2012). Anodic Nb_2_O_5_ nonvolatile RRAM. IEEE Trans. Electron Devices.

[26] Aglieri V., Zaffora A., Lullo G., Santamaria M., Di Franco F., Lo Cicero U., Mosca M., Macaluso R. (2018). Resistive switching in microscale anodic titanium dioxide-based memristors. Superlattices Microstruct..

[27] Zrinski I., Mardare C.C., Jinga L.I., Kollender J.P., Socol G., Minenkov A., Hassel A.W., Mardare A.I. (2021). Electrolyte-dependent modification of resistive switching in anodic hafnia. Nanomaterials.

[28] Zrinski I., Löfler M., Zavašnik J., Cancellieri C., Jeurgens L.P.H., Hassel A.W., Mardare A.I. (2022). Impact of Electrolyte Incorporation in Anodized Niobium on Its Resistive Switching. Nanomaterials.

[29] Aziz J., Kim H., Rehman S., Hur J.H., Song Y.H., Khan M.F., Kim D. (2021). kee Effect of oxygen stoichiometry on the threshold switching of RF-sputtered NbOx (x = 2.0–2.5) films. Mater. Res. Bull..

[30] Park K., Ryu J., Sahu D.P., Kim H.M., Yoon T.S. (2022). Electroforming-free threshold switching of NbOx-based selector devices by controlling conducting phases in the NbOx layer for the application to crossbar array architectures. RSC Adv..

[31] Kang X., Li Y., Zhu M., Jin R. (2020). Atomically precise alloy nanoclusters: Syntheses, structures, and properties. Chem. Soc. Rev..

[32] Sun B., Guo T., Zhou G., Ranjan S., Jiao Y., Wei L., Zhou Y.N., Wu Y.A. (2021). Synaptic devices based neuromorphic computing applications in artificial intelligence. Mater. Today Phys..

[33] Ryu J.H., Mahata C., Kim S. (2021). Long-term and short-term plasticity of Ta_2_O_5_/HfO_2_ memristor for hardware neuromorphic application. J. Alloys Compd..

[34] Gao B., Bi Y., Chen H.Y., Liu R., Huang P., Chen B., Liu L., Liu X., Yu S., Wong H.S.P. (2014). Ultra-low-energy three-dimensional oxide-based electronic synapses for implementation of robust high-accuracy neuromorphic computation systems. ACS Nano.

[35] Zrinski I., Minenkov A., Cancellieri C., Hauert R., Mardare C.C., Kollender J.P., Jeurgens L.P.H., Groiss H., Hassel A.W., Mardare A.I. (2022). Mixed anodic oxides for forming-free memristors revealed by combinatorial screening of hafnium-tantalum system. Appl. Mater. Today.

[36] Borghetti J., Snider G.S., Kuekes P.J., Yang J.J., Stewart D.R., Williams R.S. (2010). Memristive switches enable stateful logic operations via material implication. Nature.

[37] Du C., Cai F., Zidan M.A., Ma W., Lee S.H., Lu W.D. (2017). Reservoir computing using dynamic memristors for temporal information processing. Nat. Commun..

[38] Zhou F., Zhou Z., Chen J., Choy T.H., Wang J., Zhang N., Lin Z., Yu S., Kang J., Wong H.S.P. (2019). Optoelectronic resistive random access memory for neuromorphic vision sensors. Nat. Nanotechnol..

[39] Mardare A.I., Ludwig A., Savan A., Wieck A.D., Hassel A.W. (2010). Combinatorial investigation of Hf-Ta thin films and their anodic oxides. Electrochim. Acta.

[40] (2006). Sodium Phosphate. Cold Spring Harb. Protoc..

[41] Zrinski I., Minenkov A., Mardare C.C., Kollender J.P., Lone S.A., Hassel A.W., Mardare A.I. (2021). Influence of electrolyte selection on performance of tantalum anodic oxide memristors. Appl. Surf. Sci..

[42] Mardare A.I., Ludwig A., Savan A., Hassel A.W. (2013). Scanning droplet cell microscopy on a wide range hafnium-niobium thin film combinatorial library. Electrochim. Acta.

[43] Mardare A.I., Ludwig A., Savan A., Hassel A.W. (2014). Properties of anodic oxides grown on a hafnium-tantalum-titanium thin film library. Sci. Technol. Adv. Mater..

[44] Mardare A.I., Savan A., Ludwig A., Wieck A.D., Hassel A.W. (2009). A combinatorial passivation study of Ta-Ti alloys. Corros. Sci..

[45] Mardare A.I., Savan A., Ludwig A., Wieck A.D., Hassel A.W. (2009). High-throughput synthesis and characterization of anodic oxides on Nb-Ti alloys. Electrochim. Acta.

[46] Mardare A.I., Yadav A.P., Wieck A.D., Stratmann M., Hassel A.W. (2008). Combinatorial electrochemistry on Al-Fe alloys. Sci. Technol. Adv. Mater..

[47] Zrinski I., Minenkov A., Mardare C.C., Hassel A.W., Mardare A.I. (2021). Composite Memristors by Nanoscale Modification of Hf/Ta Anodic Oxides. J. Phys. Chem. Lett..

[48] Lanza M., Wong H.S.P., Pop E., Ielmini D., Strukov D., Regan B.C., Larcher L., Villena M.A., Yang J.J., Goux L. (2019). Recommended Methods to Study Resistive Switching Devices. Adv. Electron. Mater..

[49] Beamson G., Briggs D. (1992). High resolution monochromated X-ray photoelectron spectroscopy of organic polymers: A comparison between solid state data for organic polymers and gas phase data for small molecules. Mol. Phys. Int. J. Interface Chem. Phys..

[50] Briggs D. (2005). X-ray photoelectron spectroscopy (XPS). Handbook of Adhesion.

[51] Barreca D., Milanov A., Fischer R.A., Devi A., Tondello E. (2007). Hafnium oxide thin film grown by ALD: An XPS study. Surf. Sci. Spectra.

[52] Zagorenko A.I., Zaporozchenko V.I., Ivanova O.P. (1992). Quantitative Auger analysis of metal oxides. Surf. Interface Anal..

[53] Mcguire G.E., Schweitzer G.K., Carlson T.A. (1973). Core electron binding energies in some Group IIIA, VB, and VIB compounds. Inorg. Chem..

[54] Nyholm R., Berndtsson A., Martensson N. (1980). Core level binding energies for the elements Hf to Bi (Z=72-83). J. Phys. C Solid State Phys..

[55] Li S., Liu X., Nandi S.K., Elliman R.G. (2018). Anatomy of filamentary threshold switching in amorphous niobium oxide. Nanotechnology.

[56] Nath S.K., Nandi S.K., Li S., Elliman R.G. (2020). Metal-oxide interface reactions and their effect on integrated resistive/threshold switching in NbOx. Nanotechnology.

[57] Aziz J., Kim H., Rehman S., Kadam K.D., Patil H., Aftab S., Khan M.F., Kim D. (2021). kee Discrete memristive levels and logic gate applications of Nb_2_O_5_ devices. J. Alloys Compd..

[58] Zrinski I., Mardare C.C., Jinga L.-I., Kollender J.P., Socol G., Hassel A.W., Mardare A.I. (2021). Phosphate incorporation in anodic hafnium oxide memristors. Appl. Surf. Sci..

[59] Mikolajick T., Wylezich H., Maehne H., Slesazeck S. (2016). Versatile resistive switching in niobium oxide. Proc.—IEEE Int. Symp. Circuits Syst..

[60] Chen A., Ma G., He Y., Chen Q., Liu C., Wang H., Chang T.C. (2018). Research on Temperature Effect in Insulator-Metal Transition Selector Based on NbOx Thin Films. IEEE Trans. Electron Devices.

[61] Kim S., Liu X., Park J., Jung S., Lee W., Woo J., Shin J., Choi G., Cho C., Park S. Ultrathin (<10 nm) Nb_2_O_5_/NbO_2_ hybrid memory with both memory and selector characteristics for high density 3D vertically stackable RRAM applications. Proceedings of the 2012 Symposium on VLSI Technology (VLSIT).

[62] Liu X., Nandi S.K., Venkatachalam D.K., Belay K., Song S., Elliman R.G. (2014). Reduced threshold current in NbO2selector by engineering device structure. IEEE Electron Device Lett..

[63] Kang M., Yu S., Son J. (2015). Voltage-induced insulator-to-metal transition of hydrogen-treated NbO_2_ thin films. J. Phys. D Appl. Phys..

[64] Rathi S., Park J.H., Lee I.Y., Baik J.M., Yi K.S., Kim G.H. (2014). Unravelling the switching mechanisms in electric field induced insulator-metal transitions in VO_2_ nanobeams. J. Phys. D Appl. Phys..

[65] Gentle A., Smith G.B. (2008). Dual metal-insulator and insulator-insulator switching in nanoscale and Al doped VO_2_. J. Phys. D Appl. Phys..

[66] Zhu S., Sun B., Ranjan S., Zhu X., Zhou G., Zhao H., Mao S., Wang H., Zhao Y., Fu G. (2019). Mechanism analysis of a flexible organic memristive memory with capacitance effect and negative differential resistance state. APL Mater..

[67] Goux L., Lisoni J.G., Jurczak M., Wouters D.J., Courtade L., Muller C. (2010). Coexistence of the bipolar and unipolar resistive-switching modes in NiO cells made by thermal oxidation of Ni layers. J. Appl. Phys..

[68] Mohammad B., Jaoude M.A., Kumar V., Al Homouz D.M., Nahla H.A., Al-Qutayri M., Christoforou N. (2016). State of the art of metal oxide memristor devices. Nanotechnol. Rev..

[69] Du H., Chen J., Tu M., Luo S., Li S., Yuan S., Gong T., Huang W., Jie W., Hao J. (2019). Transition from nonvolatile bipolar memory switching to bidirectional threshold switching in layered MoO_3_ nanobelts. J. Mater. Chem. C.

[70] Ma Y., Yeoh P.P., Shen L., Goodwill J.M., Bain J.A., Skowronski M. (2020). Evolution of the conductive filament with cycling in TaOx-based resistive switching devices. J. Appl. Phys..

[71] Kim G.S., Park T.H., Kim H.J., Ha T.J., Park W.Y., Kim S.G., Hwang C.S. (2018). Investigation of the retention performance of an ultra-thin HfO_2_ resistance switching layer in an integrated memory device. J. Appl. Phys..

