# Supplementary material for: Threshold Switching in Forming-Free Anodic Memristors Grown on Hf–Nb Combinatorial Thin-Film Alloys
Source: Nanomaterials (Basel). 2022 Nov 9;12(22):3944. doi: 10.3390/nano12223944 (PMC9697845; doi:10.3390/nano12223944)
Supplement: Supplementary file 1 [file nanomaterials-12-03944-s001.zip › nanomaterials-2016487-supplementary.pdf]

Supporting Information

# Threshold Switching in Forming-Free Anodic Memristors Grown on Hf–Nb Combinatorial Thin-Film Alloys

**Ivana Zrinski [1], Janez Zavašnik [2], Jiri Duchoslav [3], Achim Walter Hassel [1,4] and Andrei Ionut Mardare [1,*]**

[1] Institute of Chemical Technology of Inorganic Materials, Johannes Kepler University Linz, Altenberger Street, 69, 4040 Linz, Austria
[2] Jožef Stefan Institute, Jamova Cesta 39, 1000 Ljubljana, Slovenia
[3] Center for Surface and Nanoanalytics, Johannes Kepler University Linz, Altenberger Street, 69, 4040 Linz, Austria
[4] Danube Private University, Steiner Landstrasse 124, 3500 Krems-Stein, Austria
* Correspondence: andrei.mardare@jku.at

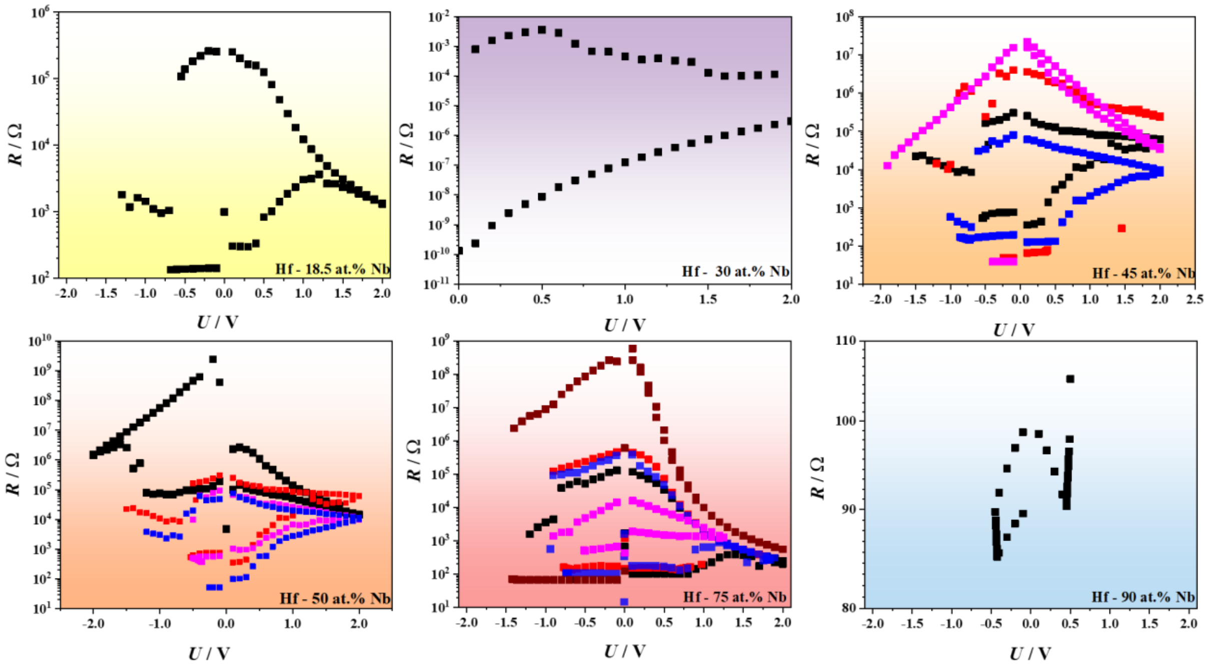


**Figure S1.** Representative *R-U* sweeps along the Hf-Nb anodic memristors.

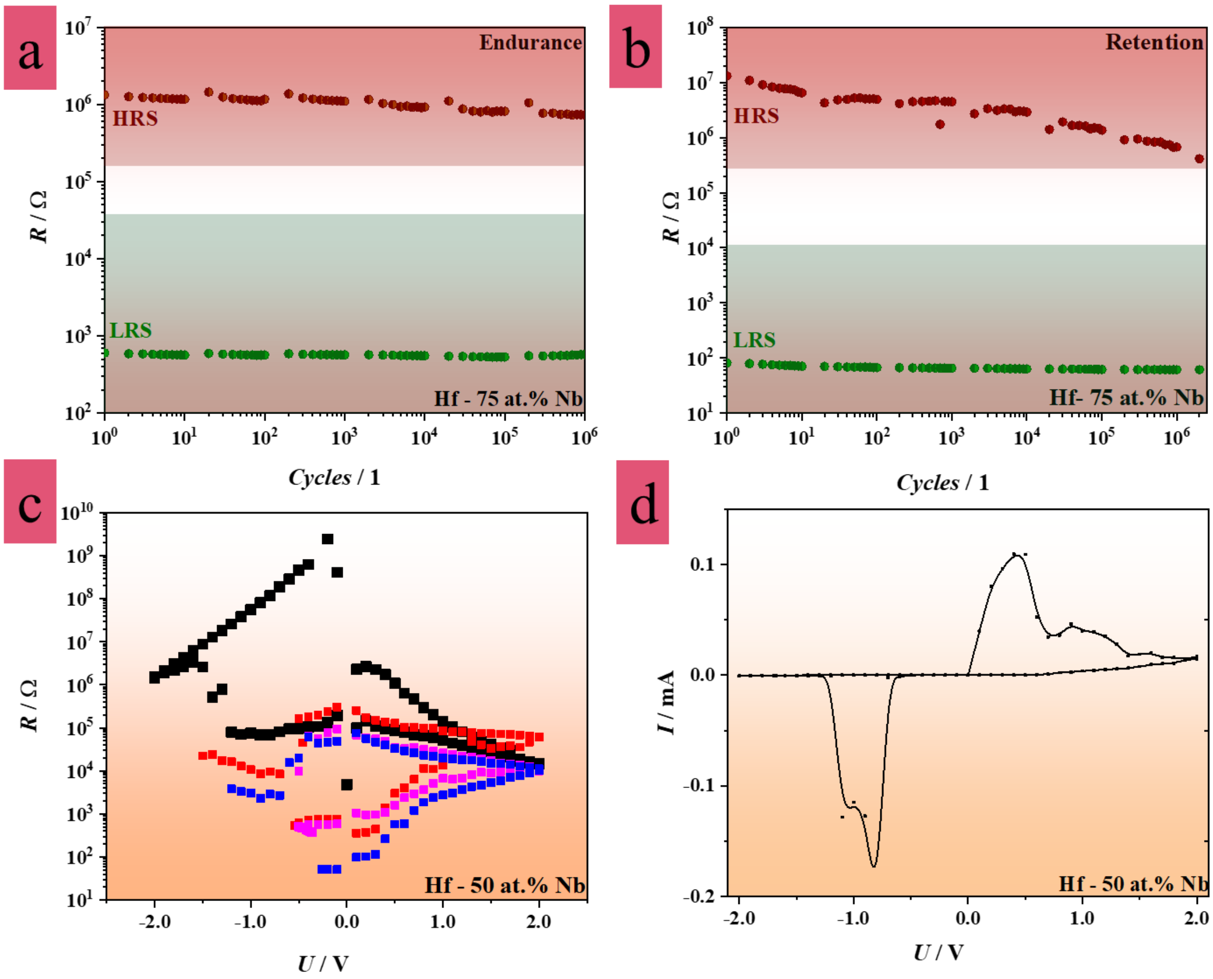


**Figure S2.** (a), (b), Endurance and retention measurements recorded for metal-insulator-metal (MIM) structures grown on Hf-75 at.%Nb alloys, (c) *R*-*U* sweeps for MIMs formed on Hf-50 at.%Nb, (d) representative *I*-*U* sweep recorded for MIMs formed on Hf-50 at.%Nb alloys.
